# Effects of Polyphenol-Rich Interventions on Cognition and Brain Health in Healthy Young and Middle-Aged Adults: Systematic Review and Meta-Analysis

**DOI:** 10.3390/jcm9051598

**Published:** 2020-05-25

**Authors:** Achraf Ammar, Khaled Trabelsi, Omar Boukhris, Bassem Bouaziz, Patrick Müller, Jordan M Glenn, Nicholas T. Bott, Notger Müller, Hamdi Chtourou, Tarak Driss, Anita Hökelmann

**Affiliations:** 1 Institute of Sport Sciences, Otto-von-Guericke University, 39104 Magdeburg, Germany; anita.hoekelmann@ovgu.de; 2 High Institute of Sport and Physical Education, University of Sfax, Sfax 3000, Tunisia; trabelsikhaled@gmail.com (K.T.); omarboukhris24@yahoo.com (O.B.); h_chtourou@yahoo.fr (H.C.); 3 Research Laboratory: Education, Motricité, Sport et Santé, EM2S, LR19JS01, High Institute of Sport and Physical Education of Sfax, University of Sfax, Sfax 3000, Tunisia; 4 Activité Physique, Sport et Santé, UR18JS01, Observatoire National du Sport, Tunis 1003, Tunisia; 5 Higher Institute of Computer Science and Multimedia of Sfax, University of Sfax, Sfax 3000, Tunisia; bassem.bouaziz@isims.usf.tn; 6 German Center for Neurodegenerative Diseases (DZNE), 39104 Magdeburg, Germany; patrick.mueller@dzne.de (P.M.); notger.mueller@dzne.de (N.M.); 7 Department of Neurology, Medical Faculty, Otto von Guericke University, 39104 Magdeburg, Germany; 8 Exercise Science Research Center, Department of Health, Human Performance and Recreation, University of Arkansas, Fayetteville, AR 72701, USA; jordan@neurotrack.com; 9 Neurotrack Technologies, 399 Bradford St, Redwood City, CA 94063, USA; nick@neurotrack.com; 10 Clinical Excellence Research Center, Department of Medicine, Stanford University School of Medicine, Stanford, CA 94305, USA; 11 Interdisciplinary Laboratory in Neurosciences, Physiology and Psychology: Physical Activity, Health and Learning (LINP2-2APS), UFR STAPS, UPL, Paris Nanterre University, 92000 Nanterre, France; tarak.driss@parisnanterre.fr

**Keywords:** cognition, neuroplasticity, brain, polyphenols, meta-analysis

## Abstract

Context: Affecting older and even some younger adults, neurodegenerative disease represents a global public health concern and has been identified as a research priority. To date, most anti-aging interventions have examined older adults, but little is known about the effects of polyphenol interventions on brain-related aging processes in healthy young and middle-aged adults. Objective: This systematic review and meta-analysis aimed to evaluate the acute and chronic effects of (poly)phenol-rich diet supplementation on cognitive function and brain health in young and middle-aged adults. In July 2019, two electronic databases (PubMed and Web of Science) were used to search for relevant trials examining the effect of acute or chronic (poly)phenol-rich supplementation on cognitive function and neuroprotective measures in young and middle-aged adults (<60 years old). A total of 4303 records were screened by two researchers using the PICOS criteria. Fifteen high quality (mean PEDro score = 8.8 ± 0.58) trials with 401 total participants were included in the final analyses. Information on treatment, study design, characteristics of participants, outcomes and used tools were extracted following PRISMA guidelines. When items were shown to be sufficiently comparable, a random-effects meta-analysis was used to pool estimates across studies. Effect size (ES) and its 95% confidence interval (CI) was calculated. The meta-analysis indicated that (poly)phenol supplementation significantly increased brain-derived neurotrophic factor (ES = 3.259, *p* = 0.033), which was accompanied by higher performance in serial (7s) subtraction (ES = 1.467, *p* = 0.001) and decreases in simple reaction time (ES = −0.926, *p* = 0.015) and mental fatigue (ES = −3.521, *p* = 0.010). Data related to cognitive function were skewed towards an effect from acute compared to chronic polyphenol intervention; data related to BDNF were skewed toward an effect from higher bioavailability phenolic components. Conclusion: This meta-analysis provides promising findings regarding the usefulness of polyphenol-rich intervention as an inexpensive approach for enhancing circulation of pro-cognitive neurotrophic factors. These beneficial effects appear to depend on the supplementation protocols. An early acute and/or chronic application of low- to high-dose phenolic components with high bioavailability rates (≥30%) at a younger age appear to provide more promising effects.

## 1. Introduction

In the last two centuries, it has been widely accepted that antibiotics and vaccines are among public health’s greatest accomplishments. Thanks to vaccination, most infectious diseases occurring in youth have been eliminated and global child mortality rates have been reduced [1]. As a result, life expectancy has increased worldwide (~27 years during the last century) with an increasing number of adults aged over 60 years old [2]. While the human race celebrates rising longevity, societies have had to confront the resulting rise of the burden of age-related global diseases [3]. It is no secret that from the fifth decade of life, advancing age is associated with an exponential increase in the accumulation of diverse deleterious changes in cells and tissues that are responsible for the occurrence of chronic diseases [4]. There is now an ongoing challenge to reduce disease burden by extending “healthspan,” thereby providing extra years spent free of chronic age-related issues such as neurodegenerative disease [5,6,7].

Affecting many older and some younger adults (≅50 million worldwide), neurodegenerative diseases (e.g., mild cognitive impairment, Alzheimer’s disease and related dementias) have become a major public health concern and have been identified as a research priority by the World Health Organisation. Unfortunately, after over 200 clinical trials, anti-aging therapies have been effective at keeping sick people alive but have failed to cure age-related neurological disorders [8]. Due to the lack of causal pharmacological treatments, brain-related “healthspan” interventions are currently directed toward slowing brain aging and cognitive decline through preventive strategies; these interventions aim to convert extra life years to health years [5,9].

The etiology of age-associated cognitive decline is complex and multifactorial with cardiovascular alteration, oxidative stress and neuroinflammation considered major risk factors [10,11,12]. The administration of exogenous antioxidants (e.g., polyphenols) has shown promising findings for reducing the majority of aforementioned risk factors [13,14,15,16,17,18,19]. Additionally, recent reports recognize (poly)phenols as a brain-friendly intervention that may prevent and delay age-associated decline in cognitive function [20,21].

These (poly)phenol compounds, such as flavonoids, phenolic acids and tannins, are found in varying concentrations in a range of plant-based food sources (e.g., legumes, fruit, vegetables, herbal extracts, spices, coffee, tea and cocoa), and their effects on human health have drawn considerable attention. Specifically, (poly)phenols have been linked to a number of health benefits including modulation of inflammation [14,15], reductions in risk of cardiovascular disease [13,15,18,22], anticancer effects [23] and protection against oxidative stress [16,17]. Concerning the neuroprotective effects, a recent systematic review (SR) and meta-analysis (MA) examining the effects of (poly)phenol-rich supplementation on age-related cognitive decline suggested polyphenol-rich supplementation may improve some cognitive and brain functions in older adults. However, it failed to provide evidence regarding the neuroprotective and anti-inflammatory effect of (poly)phenol supplementation in aging adults [20]. Findings from some individual studies, included in this SR, indicate that polyphenol consumption modulates cerebral hemodynamics [24] and resting regional cerebral blood flow [25,26], while simultaneously enhancing psychomotor functions, speed of attention, episodic memory, verbal fluency and overall cognitive performance in older-aged adults [24,27,28]. Other studies in similar populations have reported nonsignificant effects on certain cognitive functions, specifically executive functioning, working memory and verbal memory [29,30], or cerebral blood flow response [31]. Authors of the previous SR and MA concluded the beneficial effect of polyphenols was dependent on ingested dose and bioavailability, suggesting more promising findings may be found in younger populations [20].

Recent reports identify young people as the most attractive targets for interventions to extend healthspan [32]. Because their organs are not yet damaged, it is theoretically possible to reduce the onset of age-related diseases and cognitive decline by applying anti-aging interventions to people while they are still young and healthy [33]. These reports suggest preventive interventions in older adults seem to be focused on the wrong end of the lifespan, which may mitigate possible beneficial effects of the tested intervention [32,34]. Yet, most human anti-aging research, including randomized trials of polyphenol interventions, examine older adults, most of them suffering from chronic disease [20,35]. As a result, very little is known about the effect of polyphenol interventions on brain-related aging processes in healthy young humans. Additionally, the few available studies in this field demonstrate controversial findings; some of them indicate improved brain function following acute and/or chronic ingestion of polyphenol-rich supplementation [36,37,38,39,40], while other findings fail to prove beneficial effects on cognitive function and brain structures of young and middle-aged adults [41,42,43].

Taken together, it seems that there are a current gap in knowledge with a lack of consensus regarding the neuroprotective effect of polyphenol intervention in healthy young individuals. Therefore, the present study aimed to systematically review the literature and conduct a MA of all trials investigating the acute and chronic effects of (poly)phenol-rich supplementation on cognitive functions and brain health in young and middle-aged healthy adults.

## 2. Method

This SR and MA was conducted and reported in accordance with the guidelines of the preferred reporting items for systematic reviews and meta-analysis (PRISMA) [44].

### 2.1. Data Sources and Search Strategy

To inform our review, a comprehensive systematic search of studies was performed electronically in PubMed and Web of Science databases from inception to July 2019. The search was limited to English language. Search strategy and combined search terms were similar to those employed by Ammar et al. [44]. The search strategies were combined, and duplicates were removed by Endnote and manually by two of the authors. Two researchers individually considered each of the located article for its appropriateness for inclusion. In case of uncertainty, discussion with a third researcher determined the final inclusion or exclusion of the article.

### 2.2. Inclusion and Exclusion Criteria

To be included in the SR, individual studies needed to fulfill the following inclusion criteria: (i) primary research published in peer-reviewed journals in English before August 2019, (ii) research conducted with healthy human adults below 55 years old (young and middle-aged adults), (iii) original studies investigating the effect of (poly)phenol-rich supplementation brain health, (iv) no severe methodological deficiencies such as absence of control comparison, participants were not blinded, or inappropriate statistical analysis procedures. Exclusion criteria were: (i) studies written in languages other than English, (ii) data from congress or workshop publications, (iii) study conducted in diseased human population or a population above 55 years old and (iv) studies in which no supplementation was administered. Findings from case studies, encyclopedias, book chapters and reviews were excluded.

### 2.3. Study Selection

**Figure 1:**
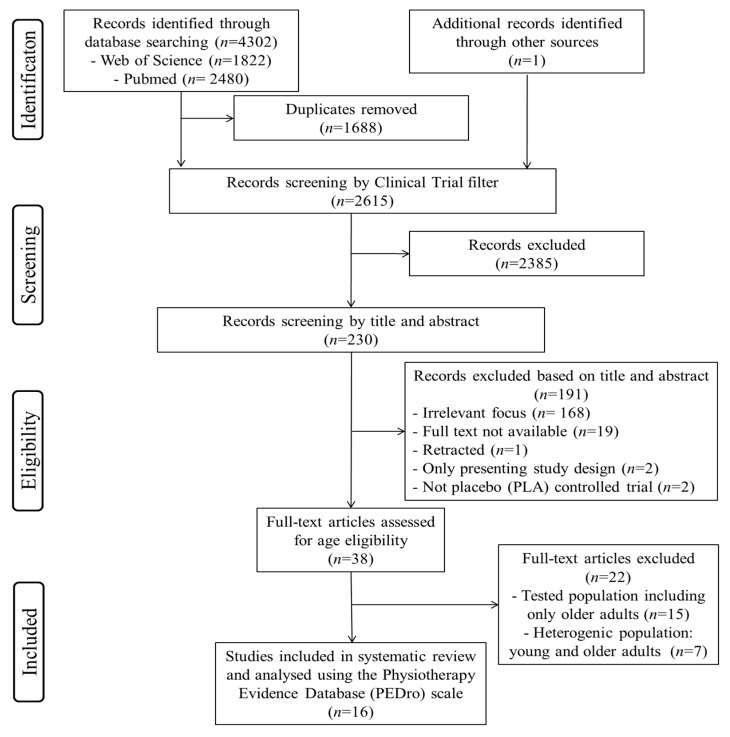
Flow diagram of the literature selection process.

Following the removal of duplicate studies from the different search engines, inclusion or exclusion of the remaining articles was performed by applying the above criteria on the title and abstract to determine eligibility in a preliminary independent screening. Selected papers were then read in full to finalize eligibility in accordance with the PICOS criteria (population: young and middle-aged adults (below 55 years); intervention: acute and/or chronic (poly)phenol-rich supplementation; comparison: any; outcome: cognitive functions (e.g., overall cognition, psychomotor performance, executive function, processing speed, attention, language, verbal memory and visual memory) and brain health measures (e.g., brain perfusion, brain activity, cerebral hemodynamics, CBF, neuroplasticity, neuroinflammation); and study design: randomized controlled trials (RCTs)). A summary of this process is outlined in Figure 1. The university’s library, hand searches, electronical databases and contact with authors were used to obtain full copies of the published manuscripts.

### 2.4. Data Extraction

Data were extracted using a standardized form. From each included study, the primary author’s first name, year of publication, study design and treatment-related data were extracted. Table 1 presents the primary outcomes related to the effects of (poly)phenol-rich supplementation on cognitive functions and Table 2 presents secondary outcomes related to the effect of (poly)phenol-rich supplementation on neuroplasticity, neuroimaging, cerebral blood flow and cerebral hemodynamics. Assessment method for each outcome was provided in specific columns within the corresponding table.

### 2.5. Quality Assessment

The Physiotherapy Evidence Database (PEDro) scale was used to assess the methodological quality of the included studies [17,20,53]. The PEDro scale is a reliable and objective tool to identify which of the RCTs are likely to be externally (criteria 1) and internally (criteria 2 to 9) valid and could have sufficient statistical information to make their results interpretable (criteria 10 and 11) [17,20,54]. Two independent reviewers assessed each paper using the 11-item checklist to yield a maximum score of 10 (the sum of awarded points for criteria 2 to 11). A consensus meeting with a third reviewer was held to resolve discrepant results. A score of 9–10 was considered to be “high quality”, 5–8 to be “moderate quality” and <5 to be “low quality” [17,20,55].

### 2.6. Statistical Analysis

The commercial software “Comprehensive Meta-Analysis” (CMA for Windows, version 3, Biostat, Englewood, USA) was utilized to conduct the MA. Given the high variability in cognitive tasks and brain measurement techniques between the included studies only simple reaction time (SRT), rapid visual information processing (RVIP), 7s serial subtraction (SS-7s), mental fatigue (MF) and brain-derived neurotrophic factor (BDNF) were shown to be sufficiently comparable and were included in the MA. To calculate the effect size, performance in reaction time (RT) was collected in seconds (s), performance in RVIP was collected in % correct, performance in SS-7s was collected in number correct, MF score was collected in scale number, and BDNF blood concentrations were collated in ng/mL. In studies where net changes were not directly reported in the intervention and control groups, the effect size (ES) was computed by subtracting the values at the endpoint of the intervention from those at baseline. The standard deviations of mean differences were calculated by using [SD = square root [(SD pre-treatment)^2^ + (SD post-treatment)^2^ − (2R × SD pre-treatment × SD post treatment)], with the correlation coefficient (R) assumed to be 0.5 [56,57]. Cohen’s method was used to calculate ES and its 95% confidence interval (CI). ES reflects the standardized difference in means (SDM) between measured parameters (i.e., RA, RVIP, SS-7s, MF and BDNF), both in response to (poly)phenol-rich supplementation and to placebo. ES was interpreted as: trivial (ES < 0.2), small (ES between 0.2 and 0.6), moderate (ES between 0.6 and 1.2), large (ES between 1.2 and 2.0), very large (ES > 2.0), and extremely large (ES > 4.0) [58]. A positive ES value in RVIP, SS-7s, and BDNF would indicate (poly)phenol-rich supplementation increased outcomes, while a negative ES in the remaining parameters (RT and MF) would indicate (poly)phenol-rich supplementation decreased outcomes. In the forest plots, the square (colored in black) represent the individual studies effect and the diamond (colored in red) represents the overall or summary effect. Q and I^2^ statistics [59,60] were used to assess statistical heterogeneity. The visual inspection of the funnel plot, the performance of the Begg and Mazumdar’s rank correlation test (Kendall’s S statistic P-Q) [61], the Egger’s linear regression test [62] and the Duval and Tweedie’s trim-and-fill test [63] were utilized to assess publication bias. Sensitivity analyses and cumulative meta-analysis were also conducted to assess the stability of and the reliability of the findings. Statistical significance was set at *p* < 0.05.

## 3. Results

Sixteen studies [36,37,38,39,40,41,42,43,45,46,47,48,49,50,51,52] examining the effects of (poly)phenol-rich supplementation on cognitive functions and/or brain related parameters were considered to meet the specific inclusion criteria and were included in the current SR.

### 3.1. Study Selection and Characteristics

#### 3.1.1. Study Selection

The predefined search strategies yielded a preliminary pool of 4303 possible papers. Removal of duplicates resulted in a selection of 2615 published papers. Removal of non-clinical trials resulted in a selection of 230 published papers. A first screening of titles and abstracts for eligibility against inclusion and exclusion criteria led to a provisional list of 38 published studies. After a careful review of the 38 full texts, 23 articles were excluded (15 studies only included older-aged adults and 7 studies investigated a heterogeneous population including both young and older adults in the same group). Therefore, 16 studies met the established inclusion criteria for determining the effects of (poly)phenol-rich supplementation on cognitive functions and a variety of neurological-related outcome measurements among aging adults. A summary of this process can be seen in Figure 1.

#### 3.1.2. Study Characteristics

The characteristics of each study, as well as the cognitive and the neurological changes following rich-(poly) phenol supplementation compared to placebo supplementation, are summarized in Table 1 and Table 2, respectively. Seven papers [36,38,45,46,47,48,50] examined the effect of rich-(poly)phenol supplementation on cognitive function (e.g., reaction time, memory, learning abilities, attention, and executive functioning), as well as a variety of brain-related parameters (e.g., neuroplasticity, cerebral hemodynamics and blood flow). Six studies [37,39,40,41,49,51] only examined the effect of rich-(poly)phenol supplementation on cognitive function. Three studies [42,43,52] only examined the change in serum BDNF [42,43] and stroke volume (SV) [52] following rich-(poly)phenol supplementation. Concerning the effect of acute and chronic (poly)phenol-rich supplementation on the above-mentioned functions, four studies investigated the chronic effect [41,42,43,45], while ten studies investigated the acute effect [37,38,39,40,46,47,48,50,51,52]. Only two studies [36,49] investigated both acute and chronic effects of (poly)phenol-rich supplementation on cognitive and brain functions.

### 3.2. Subject Characteristics

A total of 401 participants were included in this SR. The number of participants in each trial ranged (i) from 12 to 48 in studies employing a within subject counterbalanced design [37,38,39,40,45,46,47,48,50,51,52], (ii) and from 16 to 60 in studies employing a parallel groups design [36,41,42,43,49]. These studies targeted a healthy young and middle-aged population with mean age ranging from 18 to 51 years.

### 3.3. Study Design and Supplement Administration

As presented in Table 1 and Table 2, all reviewed studies employed a randomized design. Five studies employed parallel experimental arms with four of them using a placebo as the control arm and rich-(poly) phenol supplementation as treatment arms [36,42,43,49], while the remaining study [41] used only poor- and rich-(poly)phenol supplementation arms. Eleven studies employed a counterbalanced crossover design with one experimental group for the different intervention arms (e.g., placebo, low dose and high dose for the majority of the study) and a wash-out period of 24 h [39], one week [38,40,46,47,48,50,51] or two weeks [37,45,52]. The majority of the included studies in this review (eleven out of sixteen) implemented a double-blind, placebo-controlled experimental design. However, two studies implemented a single-blind, placebo-controlled experimental design [38,39]. The three remaining studies focused on the effect of different doses of (poly)phenol supplementation without using a placebo control. Two studies implemented a double-blind design [41,45] and one study implemented a single-blind design [52]. The sixteen trials included in this review employed different varieties of dietary (poly)phenol supplementation with an intervention period that ranged from acute (up to 6 h) to multiple days/weeks (e.g., 5 days to 10 weeks). Three studies opted for resveratrol extract treatment with a dose ranging from 250 mg to 500 mg in the study of Kennedy et al. [46], or a daily trans-resveratrol dose of 500 mg in the study of Wightman et al. [36], or a dose of 250 mg resveratrol alone or in combination with 20 mg piperine as reported in the study of Wightman et al. [48]. Four studies opted for rich-flavanol cocoa treatment that allowed a daily flavanol dose of 150 mg in the study of Francis et al. [45], or a dose of 250 mg or 900 mg cocoa flavanols as reported in the studies of Massee et al. [49] and Decroix et al. [50], respectively, or a dose ranging from 374 mg (low dose) to 747 mg (high dose) as reported in the study of Karabay et al. [40]. Three studies opted for oral ingestion of the ‘green tea’ polyphenol epigallocatechin gallate (EGCG) with a dose ranging from 135 mg to 270 mg [47], or a matcha tea with a dose of 4 g [39], or green tea extract (GTE) supplementation with a dose of 250 mg combined with CrossFit-style workouts [43]. The six remaining studies opted for soya extract treatment with a daily dose of 100 mg [41], or a drink of wine and ethanol [52], or a flavonoid-rich orange juice with a dose of 272 mg [37], or a dose of 500 mL flavanone-rich citrus juice containing 70.5-mg flavonoids [38], or a dose of 230 mL purple grape juice [51], or a daily dose of 160 mg of ginkgo biloba supplementation [42]. Additionally, different cognitive test batteries such as Swinburne University Computerized Cognitive Assessment Battery (SUCCAB) (Massee et al., 2015) [49], Cognitive Demand Battery (CDB) [49], the computerized battery of cognitive tasks (the Computerized Mental Performance Assessment System) [47,48,51], the Stroop task [50], the 45-min cognitive battery [38], the Cognitive Drug Research battery (CDR) [39], or a combination of validated cognitive tests [37,40,41,45,46] have been employed to assess the effect of (poly)phenol-rich supplementation on a variety of cognitive functions in a young and middle-aged population (Table 1). Similarly, different neuroimaging techniques such as near-infrared spectroscopy (NIRS) [36,46,47,48,50], biochemical analysis (BDNF) [42,43,50], FMRI [38,45], and transcranial Doppler (TCD) ultrasonography [36,49,52] have been employed to assess the effect of polyphenol-rich supplementation on a variety of neurological functions in aging populations (Table 2).

### 3.4. Methodological Quality of Studies

Overall, the study quality was deemed to be good to excellent (Table 3). The PEDro scale revealed a high score of seven and above for all included studies (mean ± SD = 8.8 ± 0.58), with 13 studies receiving a very high score of 9 out of 10 (i.e., a double-blind but not triple-blind trial). A score of 8 was given to two investigations [38,39] (i.e., no concealed allocation and no triple-blind trial) and a score of 7 was given to one investigation [52] as the authors failed to blind the therapists and investigators and to conceal allocation. 

### 3.5. Effect of (poly)Phenol Rich Supplementation on Cognitive Functions

Of the 16 studies included in this MA, 13 assessed the acute and/or chronic effect of (poly)phenol-rich supplementation on cognitive functions in a young and middle-aged population (Table 1). Five studies showed no significant effect of acute [46,47,48,50] or chronic [45] (i.e., 5 days) administration of (poly)phenol-rich supplementation (i.e., 150 mg (Francis et al., 2006) or 900 mg (Decroix et al., 2016) of cocoa flavanols or 135–500 mg of trans-resveratrol [46,47,48]) on cognitive functions. Three studies showed a significant improvement of only one cognitive function out of the overall tested cognitive functions following the chronic (28 days) and/or acute consumption of (poly)phenol-rich supplementation. Particularly, 28-day supplementation of 500 mg of trans-resveratrol results in more correct 3-Back responses with no beneficial effect on serial subtraction and rapid visual information processing (RVIP) [36]. Furthermore, 500-mL citrus juice containing 70.5 mg flavonoids significantly improved performance on the Digit Symbol Substitution Test at 2 h relative to baseline and the control drink, but no effects were observed on any other behavioral cognitive tests [38]. A dose of 300 mL of cocoa flavanols improved visual search efficiency, reflected by reduced reaction time without facilitating temporal attention nor integration [40]. Three studies showed that chronic (4 to 10 weeks) and/or acute consumption of (poly)phenol-rich supplementation resulted in a significant improvement on at least two cognitive functions. Particularly, File et al. [41] showed a daily consumption of high soya (100 mg total isoflavones/day) diet for 10 weeks may improve short-term (immediate recall of prose and 4-s delayed matching to sample of patterns) and long-term memory (picture recall after 20 min) as well as mental flexibility (rule shifting and reversal) in males and females, with an improvement of performance in a letter fluency test and in a test of planning (Stockings of Cambridge) only in females. There were no effects in tests of attention or in a category generation task. Massee et al. [49] showed that a 250 mg dose of cocoa flavanol reduced participants’ self-reported mental fatigue and improved minor aspects of cognitive performance acutely, but not sub-chronically during a highly demanding task, with no significant effects on cognition measured with the SUCCAB. Dietz et al. [39] showed significant improvements in tasks measuring basic attention abilities and psychomotor speed in response to stimuli over a defined period of time following consumption of 4 g matcha tea, with no effect in other tasks of the CDR test battery. The remaining two studies showed either an improvement in overall cognitive z-score and subjective alertness following 200 mL flavonoid-rich orange juice [37], or an improvement of overall speed on attention tasks following purple grape juice [51].

### 3.6. Effect of (poly)Phenol Rich Supplementation on Brain Parameters

A total of ten studies assessed the acute and/or chronic effect of (poly)phenol-rich supplementation on brain parameters in a young and middle-aged population (Table 2). Five studies focused on cerebral blood flow (CBF) or hemodynamics, as indexed by concentration changes in oxygenated and deoxygenated hemoglobin, showing a significant acute effect with (i) higher values following the consumption of 250 or 500 mg of trans-resveratrol [36,46,48], or 900 mg cocoa flavanol [50] and (ii) lower values following 135 mg EGCG [47]. However, chronic ingestion of 900 mg cocoa flavanol showed no effect on CBF at day 28 [36]. Three studies investigated the effect of (poly)phenol-rich supplementation (i.e., 900 mg cocoa flavanol [50]; 160 mg ginkgo biloba/day [42], 500 mg green tea extract [42,43]) on neuroplasticity, but failed to show a significant acute or delayed effect on BDNF. Two studies focused on fMRI assessment demonstrating that 5 days of cocoa flavanols (150 mg/day) supplementation increases the BOLD signal intensity in response to a cognitive task and the cerebral blood flow to gray matter [45] and that 500-mL citrus juice containing 70.5 mg flavonoids increases regional perfusion in the inferior and middle right frontal gyrus at 2 h compared to baseline and the control drink [38]. The remaining study assessing the stroke volume (SV) using Doppler ultrasound directed above the aortic annulus showed SV was higher after two drinks of red wine than after two drinks of water with no difference between the acute effects of ethanol and red wine.

### 3.7. Meta-Analysis Results

#### 3.7.1. Simple Reaction Time

Data from five studies [39,40,45,49,51] (comprising 141 participants) investigating the effect of (poly)phenol-rich supplementation on simple reaction time were pooled in our MA. Because the studies of Karabay et al. [40] included two doses of polyphenols, and because the study of Massee et al. [49] included two intervention periods, results from each condition were considered as independent studies.

**Figure 2:**
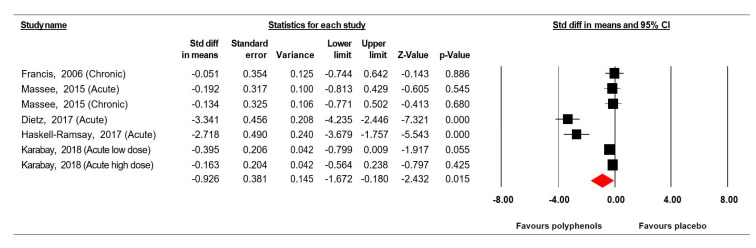
Forest plot of studies investigating the effect of (poly) phenols-rich supplementation on simple reaction time (SRT).

The summarized effects of seven ESs showed a moderate effect (ES = −0.926, SE = 0.381, 95% CI −1.672 to −0.180, Z-value = −2.432, *p* = 0.015; Figure 2) of (poly)phenol-rich supplementation on simple reaction time. A significant heterogeneity (Q = 65.955, df = 6, *p* = 0.000; *I^2^* = 90.903%) was reported.

**Figure 3:**
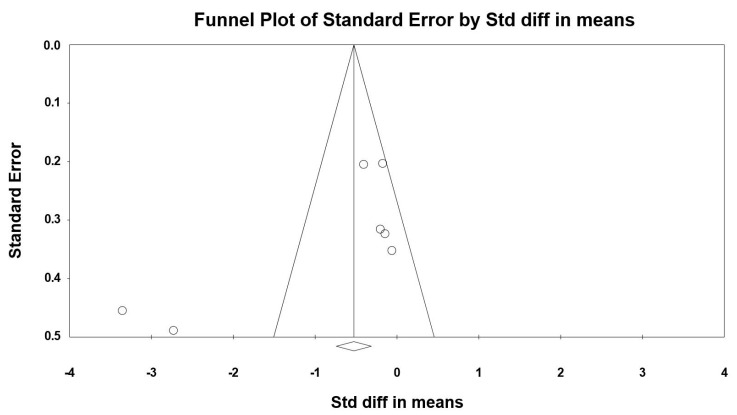
Funnel plot for SRT showing evidence of publication bias.

Visual inspection of the funnel plot (Figure 3) and the performance of the Egger’s linear regression test (intercept = −6.869, SE = 3.193, 95% CI −15.076 to 1.339, *t* = 2.151, df = 5, *p* = 0.042) showed evidence of publication bias. However, the Begg and Mazumdar’s rank correlation test (Kendall’s S statistic P-Q = −7.00; tau without continuity correction = −0.333, z = 1.051, *p* = 0.147; tau with continuity correction = −0.286, z = 0.901, *p* = 0.184) showed the lack of publication bias. The Duval and Tweedie’s trim-and-fill test confirms the absence of publication bias.

#### 3.7.2. Rapid Visual Information Processing (% correct)

**Figure 4:**
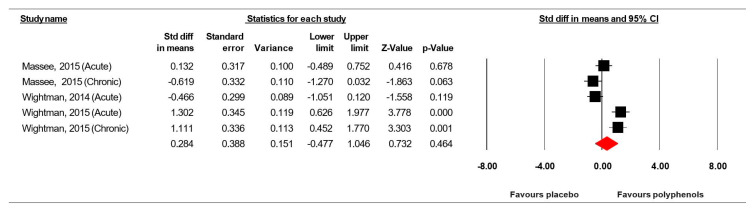
Forest plot of studies investigating the effect of (poly) phenols-rich supplementation on rapid visual information processing (% correct) (RVIP).

Data from three studies [36,48,49] (comprising 121 participants) investigating the effect of (poly)phenol-rich supplementation on rapid visual information processing were pooled in our MA. Because the studies of Massee et al. [49] and Wightman et al. [36] included two intervention periods, results from each condition were considered as independent studies. The summarized effects of five ESs showed a small effect (ES = 0.284, SE = 0.388, 95% CI −0.477 to 1.046, Z-value = 0.738, *p* = 0.464; Figure 4) of (poly) phenol-rich supplementation on the rapid visual information processing. The statistical heterogeneity was high (Q = 28.572, df = 4, *p* = 0.000; I^2^ = 86.00%).

**Figure 5:**
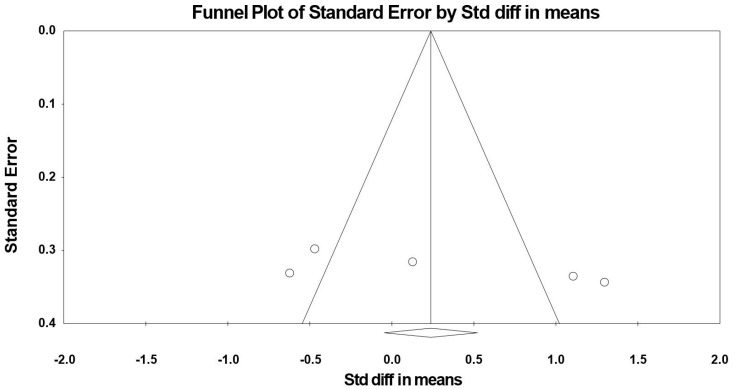
Funnel plot for RVIP showing no evidence of publication bias.

The funnel plot (Figure 5) showed no evidence of publication bias. The Begg and Mazumdar’s rank correlation test (Kendall’s S statistic P-Q = 6.00; tau without continuity correction = 0.60, *z* = 1.470, *p* = 0.071; tau with continuity correction = 0.50, *z* = 1.225, *p* = 0.110), the Egger’s linear regression test (intercept = 32.153, SE = 19.481, 95% CI −29.843 to 94.194, *t* = 1.650, df = 3, *p* = 0.098) and the Duval and Tweedie’s trim-and-fill test confirmed the lack of publication bias.

#### 3.7.3. Mental Fatigue

Data from three studies [36,48,49], comprising 121 participants, investigating the effect of (poly) phenol-rich supplementation on mental fatigue were pooled in our MA.

**Figure 6:**
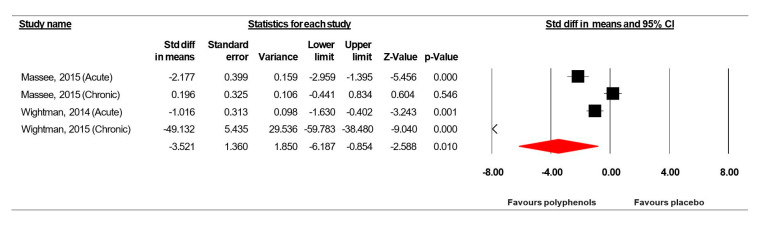
Forest plot of studies investigating the effect of (poly) phenols-rich supplementation on mental fatigue (MF).

Because the studies of Massee et al. [49] included two intervention periods, results from each condition were considered as independent studies. The summarized effects of three ESs showed a very large effect (ES = −3.521, SE = 1.360, 95% CI -6.187 to −0.854, Z-value = −2.588, *p* = 0.010; Figure 6) of (poly)phenol-rich supplementation on mental fatigue. A significant heterogeneity (Q = 100.496, df = 3, *p* = 0.000; *I^2^* = 97.015%) was computed.

**Figure 7:**
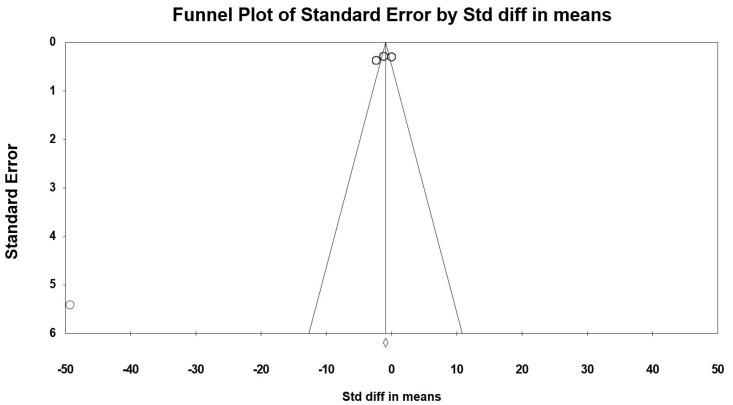
Funnel plot for MF showing evidence of publication bias.

Visual inspection of the funnel plot (Figure 7) and the performance of the Egger’s linear regression test (intercept = −9.801, SE = 2.689, 95% CI −21.369 to 1.767, t = 3.645, df = 2, *p* = 0.034) showed evidence of publication bias. However, the Begg and Mazumdar’s rank correlation test (Kendall’s S statistic P-Q = −4.00; tau without continuity correction = −0.667, *z* = 1.359, *p* = 0.087; tau with continuity correction = −0.50, *z* = 1.019, *p* = 0.154) showed no evidence of publication bias. With the Duval and Tweedie trim-and-fill analysis, one study [36] was trimmed, resulting in a “true ES” of −10.475.

#### 3.7.4. Serial Sevens Subtraction Task (Correct in Number)

**Figure 8:**
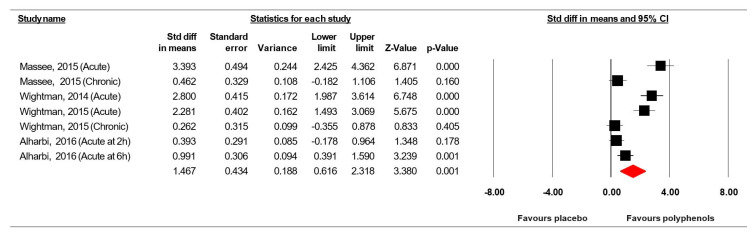
Forest plot of studies investigating the effect of (poly) phenols-rich supplementation on Serial Sevens subtraction task (correct in number) (SS-7s).

Data from four trials [36,37,48,49] (comprising 145 participants) investigating the effect of (poly)phenol-rich supplementation on Serial Sevens subtraction task were pooled in our MA. Because the studies of Massee et al. [49], Wightman et al. [36] and Alharbi et al. [37] included two intervention periods or two assessment times, results from each condition were considered as independent studies. The summarized effects of three ESs showed a moderate effect (ES = 1.467, SE = 0.434, 95% CI 0.616 to 2.318, Z-value = 3.380, *p* = 0.001; Figure 8), with a significant heterogeneity (Q = 63.471, df = 6, *p* = 0.000; *I^2^* = 90.587%).

**Figure 9:**
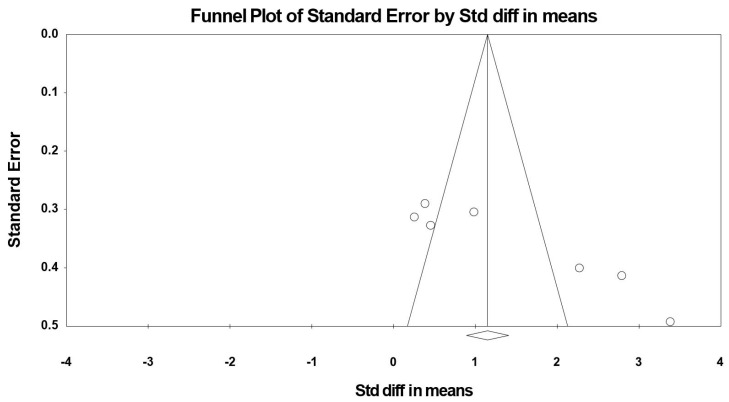
Funnel plot for SS-7s showing evidence of publication bias.

Visual inspection of the funnel plot (Figure 9), the performance of the Egger’s linear regression test (intercept = 16.711, SE = 2.751, 95% CI 9.640 to 23.782, t = 6.075, df = 5, *p* = 0.001) and the Begg and Mazumdar’s rank correlation test (Kendall’s S statistic P-Q = 15.00; tau without continuity correction = 0.714, *z* = 2.253, *p* = 0.012; tau with continuity correction = 0.667, *z* = 2.103, *p* = 0.018) showed evidence of publication bias. However, the Duval and Tweedie trim-and-fill analysis did not identify any study to trim.

#### 3.7.5. Brain-Derived Neurotrophic Factor

Data from three trials [42,43,50] (comprising 46 participants) investigating the effect of (poly)phenol-rich supplementation on brain-derived neurotrophic factor (BDNF) were pooled in our MA.

**Figure 10:**
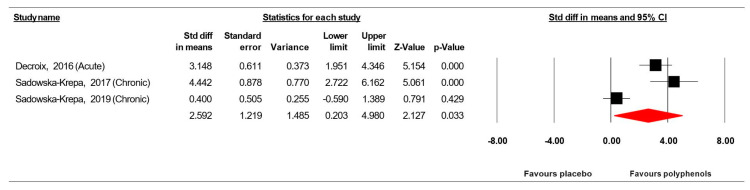
Forest plot of studies investigating the effect of (poly) phenols-rich supplementation on brain-derived neurotrophic factor (BDNF).

**Figure 11:**
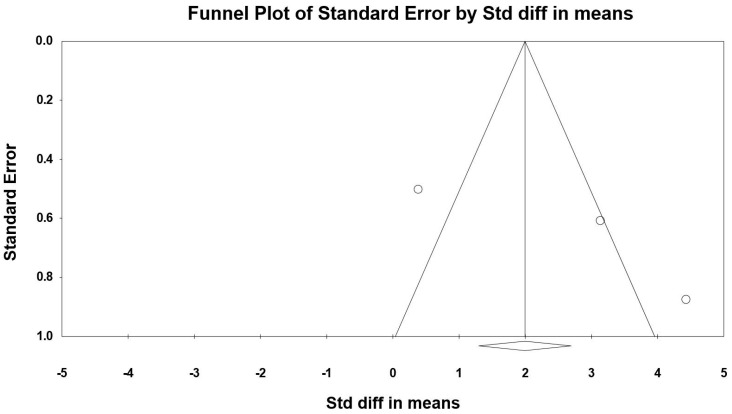
Funnel plot for BDNF showing no evidence of publication bias.

The summarized effects of three ESs showed a very large effect (ES = 3.259, SE = 1.219, 95% CI 0.203 to 4.980, Z-value = 2.127, *p* = 0.033; Figure 10) of (poly)phenol-rich supplementation on BDNF. There was a significant heterogeneity (Q = 21.318, df = 2, *p* = 0.000; *I^2^* = 90.618%). No evidence of publication bias was detected after the visual inspection of the funnel plot (Figure 11). This conclusion was confirmed by the Begg and Mazumdar’s rank correlation test (Kendall’s S statistic P-Q = 3.00; tau without continuity correction = 1.00, *z* = 1.567, *p* = 0.059; tau with continuity correction = 0.667, *z* = 1.045, *p* = 0.148), the Egger’s linear regression test (intercept = 11.122, SE = 5.771, 95% CI −62.610 to 84.454, *t* = 1.927, df = 1, *p* = 0.152) and the Duval and Tweedie’s trim-and-fill test.

### 3.8. Sensitivity and Cumulative Meta-Analysis

In summary, the reliability and stability of the findings were confirmed by the sensitivity and cumulative meta-analysis.

## 4. Discussion

The present SR and MA is the first to examine the effects of acute and chronic (poly)phenol-rich supplementation on cognitive and brain parameters in young and middle-aged adults. Data regarding changes in a variety of cognitive functions and brain parameters following an acute and/or chronic consumption of (poly)phenol-rich supplementation were extracted from the reviewed trials. However, only a few items were sufficiently comparable and were included in the MA (i.e., SRT, RVIP, SS-7s, MF and BDNF). The pooled analysis of the acute and/or chronic administrations (4 weeks) of (poly)phenol-rich supplementation suggests a beneficial effect on the majority of the assessed cognitive functions including SRT, SS-7s and MF with faster SRT, higher correct numbers during SS-7s and lower MF compared to placebo condition. Particularly, the data of the forest plots are significantly skewed towards an effect from acute compared to chronic polyphenol intervention. For the SS-7s cognitive test, a significant effect was observed for the acute administration of 500mg trans-resveratrol [36], 272 mg flavonoids [37], and 250 mg catechin [49] or resveratrol [48]. However, no significant effects were observed for the chronic administration (4 weeks) of similar catechin [49] and trans-resveratrol [36] doses. Similarly, a significant beneficial effect on MF was observed for the acute administration of 250 mg polyphenols [48,49]. However, this effect was blinded during the chronic administration of the same dose [49]. Moreover, the two effective (poly)phenols doses (300 mg of phenolic contents [51] or 4 g of matcha tea [39]) on SRT were isolated only to acute administration. These results indicate a beneficial effect of an acute (poly)phenol-rich supplementation on the majority of the assessed cognitive functions and suggest an acute dose of 250 mg (poly)phenols is sufficient to generate an immediate improvement in SS-7s and MF, while a higher dose is needed to observe a significant effect on SRT. Similarly, a chronic dose of 250–500 mg (poly)phenol showed no significant effect on cognitive functions [36,49]; it seems that higher doses (>500 mg/day) and/or higher bioavailability of phenolic contents are needed during chronic interventions to improve cognitive functions.

In agreement with these findings, some of the included studies in the MA have reported that an acute ingestion of 250–300 mg of cocoa flavanols improved visual search efficiency and aspects of cognitive performance during a highly demanding task and reduced reaction time and participants’ self-reported mental fatigue [40,49]. Similarly, in response to an acute dose of 4 g matcha tea or 200 mL of purple grape juice, Dietz et al. [39] and Haskell-Ramsay et al. [51] demonstrated a significant improvement in tasks measuring basic attention abilities and psychomotor speed. Besides, the improvement of these specific cognitive functions and findings from other individual studies confirm the beneficial effect of both acute and chronic consumption of (poly)phenol-rich supplementation on further cognitive performances and showed improved performance on the digit symbol substitution test at 2 h following an acute consumption of 500 mL of citrus juice containing 70.5 mg flavonoids [38]. Regarding chronic (poly) phenol-rich supplementation, only one study showed a significant improvement on multiple cognitive functions including short- and long-term memory, mental flexibility, planning and letter fluency [41] following 10 weeks of a daily dose of 100 mL of isoflavone with high phenolic bioavailability (≈43%).

The exact mechanism behind the beneficial action of short-term (poly)phenol supplementation in relation to cognition is yet to be conclusively determined. However, a number of potential direct and indirect mechanisms have been proposed to explain the beneficial effects of phenolic compounds on brain function [64,65]. These mechanisms include:

(i) interaction with gut microbiota [66] which is known to impact (poly)phenol absorption [67,68],
(ii) modulation of neuroinflammation [69] and glucoregulation [70] with previous studies have demonstrated that impaired glucose tolerance is associated with poorer cognition [71], improved cerebrovascular function (e.g., CBF, [72]),
(iii) and increased spine density and neurogenesis, particularly in the hippocampus [73].

Given the multifunctional nature of (poly)phenol effects, it was recently suggested that that all of these mechanisms have a role to play and are also interrelated [40,51] with endothelial nitric oxide (NO) representing a key molecule in this relationship [74].

Because NO has multiple biological functions, previous studies have reported that the physiological beneficial effects of (poly)phenol likely depend in part on its ability to: promote NO synthesis, contribute to flow-mediated dilation [75], and enhance nitric oxide synthase (NOS) activity as well as NO bioavailability (through limiting NO scavenging by ROS [76]). Particularly, enhanced cognition due to (poly)phenol consumption is widely reported to be caused by two main effects: NO synthesis and vasodilation and neurotransmission [40,77]. Indeed, by stimulating the guanylate cyclase, NO systems mediate vasodilation in blood vessels including cerebral arteries [78], which results in increased CBF parameters. Consistent findings from several individual studies, including those in the present SR, confirm enhanced cognitive performance in healthy young adults is accompanied by an increase in CBF or cerebral blood oxygenation following the consumption of 250 or 500 mg of trans-resveratrol [36,46,48] or high flavanol cocoa drink [45,50]. Similarly, consumption of 500-mL citrus juice containing 70.5 mg flavonoids increases regional perfusion in the anterior cingulate cortex and central opercular cortex of the left parietal lobe at 2 h post consumption compared to the control drink [38]. As the anterior cingulate cortex is involved in attention and executive function modulation [79], the confirmed cognitive improvement (e.g., SRT) following (poly)phenol ingestion may be related to the ability of its components to activate NO synthesis responsible for vasodilation and increased activity in this region. However, because vasodilation is not the only relevant biological role of NO, it cannot be assumed that increasing regional perfusion is solely responsible for improved cognitive performance. Independent of its CBF effects, via the activation of NO synthesis, (poly)phenols also influence neuronal signaling pathways [77], as NO acts as a neurotransmitter [80]. This offers an alternative explanation of the enhanced cognition following (poly)phenol consumption. Taken together, it is likely that the positive effect of (poly)phenols on brain health is mainly due to two principal effects of the activated NO synthesis pathways: vasodilation and neurotransmission. Nevertheless, because our MA showed that rich (poly)phenol supplementation enhances the majority (SRT, SS-7s and MF), but not all, of cognitive functions (i.e., no effect on RVIP), it appears that the modulations of cognitive functions in response to polyphenol supplementation are more related to neurotransmission rather than vasodilation [40]. Indeed, if increased vasodilation and CBF are the causal factors, then beneficial effects of (poly)phenols should appear in all cognitive functions and should not be dependent on the type of cognitive process measured, which was not the case in our MA (pooled data for RVIP revealed a non-significant effect).

Regarding the effect of (poly)phenol supplementation on neuroplasticity biomarkers, while individual studies failed to show significant improvement following the consumption of 900 mg cocoa flavanol [50], 160mg rich-flavonoid Ginkgo biloba [42] or 250 mg rich-catechin green tea extract [43], pooled analyses suggest a significant effect on BDNF with higher values compared to placebo condition.

It is well documented that enhanced cognitive functions are related to an increase in serum BDNF levels in the brain stimulating synaptic plasticity and neurogenesis [81] and that BDNF plays an important role in learning and memory functions [82]. The pooled findings of the present MA support these reports and show that the significant beneficial effect of (poly)phenol supplementation on BDNF was accompanied by improved cognitive function including SS-7s, MF and SRT.

Previous reports also indicate that: (i) natural catechin polyphenol can be associated with an increased expression of BDNF and higher cognitive function [83]; (ii) green tea polyphenols can boost the neuritogenic activity BDNF through the activation of NADPH-oxidase pathway [84]; and (iii) flavonoids, at low nanomolar concentrations, also induce synaptic plasticity [85] via modulation of receptor function, gene expression and interaction with signaling pathways [86]. The present MA supports the majority of these findings and pooling the findings related to the effect of different polyphenol supplementation [42,43,50] indicate an increase in BDNF levels compared to placebo.

Particularly, the forest plots reveal (i) a significant effect of an acute dose of 900mg cocoa flavanol [50], (ii) a significant effect of a chronic (6 weeks) daily dose of ≈40 mg flavonoid with ≈30% phenolic bioavailability [42] and (iii) a non-significant effect of a chronic (6 weeks) daily dose of 400 mg catechin with ≈18% phenolic bioavailability [43]. These results indicate that data related to chronic (poly)phenol interventions are skewed towards a beneficial effect on BDNF from higher phenolic bioavailability components. These findings support a recent suggestion [20] indicating that health effects of (poly)phenols on brain plasticity are closely associated with their bioavailability. Indeed, (poly)phenol components with high bioavailability can cross the blood–brain barrier [87] and interact with the cellular cascade resulting in upregulation of brain BDNF gene or protein expression [88].

A recent SR and MA conducted by our research team and addressing cognition and brain function in the elderly population failed to provide evidence regarding the beneficial effect of acute and/or chronic rich (poly)phenol supplementation on executive function, brain plasticity and inflammatory markers [20]. By showing a significant beneficial effect of similar supplementation on brain plasticity biomarkers (i.e., acute and chronic interventions) and on different cognitive functions (i.e., specifically acute intervention) in young and middle- aged adults, the present paper supports the recent theory identifying young people as the most attractive targets for intervention to extend healthspan [32]. Indeed, because their brain’s organs are not yet damaged, it seems possible that anti-aging interventions targeting young and healthy people will better prevent onset of age-related diseases and cognitive decline [33]. However, since chronic studies in young and middle-aged adults are quite short (the majority are between 4 and 10 weeks) when compared with older-adult studies (the majority are 12 weeks with some lasting up to 6 months), further meta-analysis and meta-regression (pooling the results of the different age groups and accounting for the intervention period) are warranted to confirm this theory.

The strengths of the present study include a comprehensive coverage of the current literature via the utilization of a wide range of key words (related to cognition and brain) searched through two scholarly databases, the focus on randomized controlled trials which are the gold standard to confirm the effects of nutritional interventions on cognitive decline, maintenance or improvement [89], and the high methodological quality (8.8) of the included studies.

However, despite its novelty, the present study is limited by (i) the relatively small sample sizes of the individual studies, which used a large variety of cognitive task batteries, imaging techniques, and brain health biomarkers, resulting in a relatively low number of included studies in the MA, (ii) the evidence of publication bias present in the mental fatigue domain, and (iii) the significant amount of heterogeneity present in all fields of the research domain; especially those related to the employed study design. Indeed, since some cognitive domains (e.g., processing speed, memory) are particularly receptive to practice effects, results must be interpreted with caution when findings from different study designs (e.g., parallel group, counterbalanced design) are pooled. Further high-quality investigations in the field are warranted.

## 5. Conclusions

This meta-analysis provides promising findings regarding the beneficial effect of (poly)phenol supplementation on cognitive functions and neuroplasticity of young and middle-aged adults. These beneficial effects appear to depend on the administration type (acute or chronic) and the supplementation protocols (dose and bioavailability) with more significant effects observed following acute supplementation. For the acute intervention, the present systematic review and meta-analysis suggests at least 250 mg (poly)phenols are required to generate an immediate improvement in SS-7s and MF, while a higher dose is needed to improve SRT (e.g., 300 mg) and BDNF (e.g., 3900 mg). For chronic interventions, higher doses (>500 mg/day) and/or higher bioavailability of phenolic contents (≥30%) are needed to generate a significant effect on cognitive functions and neuroprotective measures. These findings provide better insight into (poly)phenols’ effect on cognitive function and neuroplasticity in young and middle-aged adults, suggesting that rich polyphenol supplementation may be highly useful as an inexpensive, long-term preventive intervention on neurodegenerative diseases and cognitive decline. However, given that beneficial effects of (poly)phenols on brain health appear to be significant among young and middle-aged adults (as evidenced by the findings from the present MA), rather than older adults [20], it is more advantageous to begin early anti-aging interventions at a younger age.

## Figures and Tables

**Table 1:** Effect of polyphenol-rich supplementation on cognitive functions in young- and middle-aged populations.

Authors	Study Design	Treatment	Phenolyc Content	Dose	Duration	Washout Period	Study Population	Effect on Cognition	Used Tools
**File et al. [41]**	Randomized, double blind, parallel-groups study	High or low soya diet	A high soya (100 mg total isoflavones/day) or a low soya (0.5 mg total isoflavones/day)	One per day	10 weeks	N/A	Twenty-seven student volunteers (15 men and 12 women)	↔ non-significant effects on tests of attention or semantic memory; ↑ significantly improve short-term and long-term memory and mental flexibility (rule shifting and reversal) in males and females; ↑ significantly improve performance in a test of planning (Stockings of Cambridge) and in a letter fluency test only in females.	The digit-symbol substitution test (DSST); The digit cancellation (DC); The paced auditory serial addition test (PASAT); test of immediate memory, a short story (from the revised Weschler Memory Scale) with 25 units of information was read at the rate of one unit per second; the Cambridge Neuropsychological Test Automated Battery (CANTAB; CeNeS Ltd., Cambridge); Long-term episodic memory was measured by presenting a set of 22 pictures of common objects – each picture was shown for 5 s and then 20 min later; Letter fluency tests; A test of rule shifting and reversal (IDED).
**Francis et al. [45]**	A double blind counterbalanced manner	Flavanol-rich cocoa	High flavanol cocoa drink (172 mg flavanols per drink), low flavanol cocoa drink (13 mg flavanols per drink)	One drink/day	5 days	2 weeks	Sixteen young female subjects between the ages of 18 and 30 years	↔ non-significant effects on behavioral reaction times and switch cost	The letter-digit task
**Kennedy et al. [46]**	Randomized, double-blind, placebo-controlled, counterbalanced order, crossover design	Resveratrol	Not specified	Two doses (250 and 500 mg)	Acute	7 days	Twenty-four healthy adults (4 men, 20 women; mean age: 20.17 years; age range: 18–25 years)	↔ non-significant effect on cognitive task performance and mental fatigue	The 9-min battery consists of 4-min Serial Subtraction, 5-min rapid visual information processing (RVIP) and a Mental Fatigue Visual Analogue Scale.
**Wightman et al. [47]**	Double-blind, placebo-controlled, counterbalanced order, crossover design	Green tea polyphenol epigallocatechin gallate (EGCG)	Two capsules each containing either 135 mg or 270 mg EGCG (94% pure EGCG plus 6% excipients)	Two doses (135 and 270 mg) of EGCG	Acute	7 days	Twenty-seven healthy adults (11 men, 16 women, mean age 22 years, range 18–30 years)	↔ non-significant effect on cognitive performance	Serial subtractions; Oddball reaction time task; rapid visual information processing task (RVIP); Stroop task; simple reaction time.
**Wightman et al. [48]**	Randomized, double-blind, placebo-controlled, counterbalanced order cross-over	Resveratrol	250 mg of trans-resveratrol	Two capsules	Acute	At least a week	Twenty-three healthy adults (four males and nineteen females, mean age 21 years, range 19–34 years, SD 3·2 years)	↔ non-significant effects on cognitive function	Serial subtractions; rapid visual information processing; N-back task.
**Massee et al. [49]**	Randomized, placebo-controlled, double-blind, parallel design	Cocoa flavanols	3058 mg T. cacao seed extract standardized to contain 250 mg catechin polyphenols and 5.56 mg caffeine	One tablet daily (250 mg)	Acute and chronic (4 weeks)	N/A	38 young, healthy participants aged 18–40 years (M = 24.13, SD = 4.47)	↑ significantly improved performance acutely on the Serial component of the Cognitive Demand Battery (CDB). ↓ significantly decrease participants’ self-reported mental fatigue prior to commencing the CDB testing battery, ↔ non-significant effects significant effects were found for cognition measured with the SUCCAB.	Swinburne University Computerized Cognitive Assessment Battery (SUCCAB) [(1) Simple reaction time; (2) Choice reaction time; (3) Immediate recognition; (4) Congruent Stroop color word; (5) Incongruent Stroop color word; (6) Spatial working memory; (7) Contextual memory; (8) Delayed recognition]; Cognitive Demand Battery (CDB) [(1) Mental fatigue scales; (2) Serial Threes subtraction task; (3) Serial Sevens subtraction task;(4) Rapid Visual Information Processing Task (RVIP); (5) Mental fatigue scales]
**Wightman et al. [36]**	Randomized, double-blind, placebo-controlled, parallel-groups study	Resveratrol	TransmaxTM by BiotiviaTM with a guaranteed purity of 98%, also containing 10 mg of piperine/capsule	500 mg once day	Acute and chronic (28 days)	N/A	Sixty adults aged between 18 and 30 years	↑ significantly improve accuracy during serial subtraction task performance as acute effect. ↑ significantly improve accuracy during the 3-Back task before treatment consumption with ↔ non-significant effect on the remaining functions.	Serial subtractions; rapid visual information processing (RVIP); 3-Back
**Alharbi et al. [37]**	Randomized, double-blind, placebo-controlled, counterbalanced order, crossover design	Flavonoid-rich orange juice	272 mg flavonoids	240-mL FR orange juice (272 mg flavonoids)	Acute	2 weeks	Twenty-four healthy males (mean age: 51 ± 6, 6 years old)	↑ significantly improve cognitive function (z score) and subjective alertness	Digit Symbol Substitution Test (seconds); Serial Sevens (number correct); Immediate Verbal Recall (words); Delayed Verbal Recall (words); Continuous Performance Task (errors); Simple Finger Tapping (correct responses); Complex Finger Tapping (correct responses); Contrast Sensitivity (Michelson Contrast)
**Decroix et al. [50]**	Randomized, double-blind, placebo-controlled, counterbalanced order, crossover design	Cocoa flavanol	High CF-content chocolate milk (CF, 903.75 mg flavanol, Acticoa) or a PLA that contained low-CF chocolatemilk (PLA, 15 mg flavanol)	900 mg	Acute	7 days	Twelve well-trained men of 30 ± 3 years old	↔ non-significant effect on cognitive performance	Reaction time (RT) and accuracy on neutral, congruent and incongruent stimuli and Stroop interference
**Lamport et al. [38]**	Randomized, single-blind, placebo-controlled, counterbalanced order, crossover design	Flavanone-rich citrus juice	70.5-mg flavonoids	500-mL citrus juice containing 70.5-mg flavonoids	Acute	1-week	Sixteen healthy young adults aged 18–30 years	↑ significantly improve performance on the Digit Symbol Substitution Test at 2 h relative to baseline and the control drink, ↔ non-significant effect on any other behavioral cognitive tests.	Freiburg Vision Test (version 3.6.3), Word Recall (immediate), Logical Memory (immediate recall), Sequence Learning Task, Digit Symbol Substitution Test (DSST), Stroop Test, Letter Memory Test, Go-No Go Task, Spatial Delayed Recall, Word Recall (delayed) and Logical Memory (delayed).
**Dietz et al. [39]**	Randomized, single-blind, placebo-controlled, counterbalanced order, crossover design	Matcha tea, matcha tea bar	4.0 g of matcha tea powder, equivalent to two average portions of matcha tea (2 × 2 g powder in 100 mL water)	4 g of matcha tea	Acute	24 h	Nineteen females and four males (mean age 24.7 years, age range 20–35 years)	↑ significantly improve tasks measuring basic attention abilities and psychomotor speed in response to stimuli over a defined period of time. ↔ non-significant effect on other tasks of the cognitive test battery.	Immediate word recall task; Simple reaction time task; Digit vigilance task; Choice reaction task; Spatial working memory task; Numeric working memory task; Delayed word recall task; Delayed word recognition task; Delayed picture recognition task; Speed of attention; Accuracy of attention; Episodic secondary memory; Working memory; Quality of memory; Speed of memory
**Haskell-Ramsay et al. [51]**	Randomized, placebo-controlled, double-blind, counterbalanced-crossover design	Purple grape juice	Phenolic content: 1504.5 μg/mL; Anthocyanin content: 138.3 mg/L	200 mL Welch’s™ purple grape juice	Acute	Between 6 and 7 days	Twenty participants (7 males; mean age 21.05 years, SD 0.89)	↑ significantly improve overall speed on attention tasks	Word presentation; Immediate word recall; Picture presentation; Simple reaction time; Digit vigilance; Choice reaction time; Numeric working memory; Delayed word recall; Delayed word recognition; Delayed picture recognition
**Karabay et al. [40]**	Randomized, double-blind, placebo and baseline-controlled counterbalanced, crossover design	Cocoa flavanols	374 mg in the low-dose condition and 747 mg in high-dose condition	300 mL	Acute	1-week	Forty-eight (24 female) healthy (mean age = 22.15 years, range = 18–29, SEM = 0.01)	↑ significantly improve visual search efficiency, reflected by reduced reaction time. ↔ non-significant effect on temporal attention nor integration	Attentional blink/integration task (RSVP); Visual search task (VS)

Abbreviations: Not applicable (N/A), epigallocatechin gallate (EGCG), placebo (PLA), cocoa flavanol (CF), digit-symbol substitution (DSS), digit cancellation (DC), Cambridge Neuropsychological Test Automated Battery (CANTAB), rapid visual information processing (RVIP), Cognitive Demand Battery (CDB), Swinburne University Computerized Cognitive Assessment Battery (SUCCAB), reaction time (RT), visual search task (VS).

**Table 2:** Effect of (poly)phenol-rich supplementation on brain health measures in young- and middle-aged population.

Authors	Study Design	Treatment	Phenolyc Content	Dose	Duration	Washout Period	Study Population	Effect on CBF	Used Techniques/Outcomes
**Francis et al. [45]**	A double blind counterbalanced manner	Flavanol-rich cocoa	High flavanol cocoa drink (172 mg flavanols per drink), low flavanol cocoa drink (13 mg flavanols per drink)	One drink/day	5 days	2 weeks	Sixteen young female subjects between the ages of 18 and 30 years	↑ significantly increase the BOLD signal intensity in response to a cognitive task, ↑ significantly increase the cerebral blood flow to gray matter	Functional magnetic resonance imaging (FMRI) based on blood oxygenation level-dependent (BOLD) contrast to explore the effect of flavanols on the human brain.
**Spaak et al. [52]**	A randomized, single-blind trial counterbalanced order, crossover design	Red wine		Dose 1 = 155 mL; dose 2 = 310 mL; given to a 68-kg man	Acute	2 weeks	Thirteen volunteers (24–47 years; 7 men, 6 women)	↑ significantly increase Stroke volume	Stroke volume (SV) was determined by Doppler ultrasound directed above the aortic annulus
**Kennedy et al. [46]**	Randomized, double-blind, placebo-controlled, counterbalanced order, crossover	Resveratrol	Not specified	Two doses (250 and 500 mg)	Acute	7 days	Twenty-four healthy adults (4 men, 20 women; mean age: 20.17 years; age range: 18–25 years)	↑ significantly increase cerebral blood flow (CBF) during task performance, as indexed by total concentrations of hemoglobin and deoxyhemoglobin	Functional NIRS is a brain-imaging technique that is predicated on the intrinsic optical absorption properties of oxygenated hemoglobin (oxy-Hb) and deoxygenated hemoglobin (deoxy-Hb) after the introduction of near-infrared light through the intact skull.
**Wightman et al. [47]**	Double-blind, placebo-controlled, counterbalanced order, crossover design	Green tea polyphenol epigallocatechin gallate (EGCG)	Two capsules each containing either 135 mg or 270 mg EGCG (94% pure EGCG plus 6% excipients)	Two doses (135 and 270 mg) of EGCG	Acute	7 days	Twenty-seven healthy adults (11 men, 16 women, mean age 22 years, range 18–30 years)	↓ significantly decrease both oxygenated and total hemoglobin, ↔ non-significant effect on deoxygenated hemoglobin.	NIRS is a non-invasive brain imaging technique in which two nominal wavelengths of light (~765 and 855 nm), which are differentially absorbed by oxygenated (oxy-Hb) and deoxygenated hemoglobin (deoxy-Hb)
**Wightman et al. [48]**	Randomized, double-blind, placebo-controlled, counterbalanced order cross-over	Resveratrol	250 mg of trans-resveratrol	Two capsules	Acute	At least a week	Twenty-three healthy adults (four males and nineteen females, mean age 21 years, range 19–34 years, SD 3·2 years, all right handed)	↑ significantly improve CBF during task performance	Near-IR spectroscopy
**Wightman et al. [36]**	Randomized, double-blind, placebo-controlled, parallel-groups study	Resveratrol	Transmax^TM^ by Biotivia^TM^ with a guaranteed purity of 98%, also containing 10 mg of piperine/ capsule	500 mg once day	Acute and chronic (28 days)	N/A	Sixty adults aged between 18 and 30 years	↑ significantly improve CBF parameters on day 1, as assessed by NIRS	Transcranial Doppler; near-IR spectroscopy (NIRS); Venous blood samples
**Decroix et al. [50]**	Randomized, double-blind, placebo-controlled, counterbalanced order, crossover design	Cocoa flavanol	High CF-content chocolate milk (903.75 mg flavanol) or a PLA contained low-CF chocolate milk (15 mg flavanol)	900 mg	Acute	7 days	Twelve well-trained men of 30 ± 3 years old	↑ significantly increase cerebral oxygenation; ↔ non-significant effect on BDNF	Functional NIRS, a noninvasive optical imaging technique, was used to assess acute changes in local cerebral blood volume (reflecting CBF) and oxygenation (Oxymon continuous-wave NIRS (CW-NIRS) system (Artinis Medical Systems B.V.); Blood parameter (BDNF)
**Lamport et al. [38]**	Randomized, single-blind, placebo-controlled, counterbalanced order, crossover design	Flavanone-rich citrus juice	70.5-mg flavonoids	500-mL citrus juice containing 70.5-mg flavonoids	Acute	1-week	Sixteen healthy young adults aged 18–30 years	↑ significantly increase regional perfusion in the inferior and middle right frontal gyrus at 2 h relative to baseline and the control drink.	fMRI arterial spin labelling (ASL)
**Sadowska-Kr˛epa et al. [42]**	Randomized, double-blind, placebo-controlled, parallel-groups study	Ginkgo biloba Supplementation	80 mg EGb capsules containing 19.2 mg flavonoid glycosides (24%)	Two capsules once a day (160 mg/day)	Six weeks	N/A	18healthy, physically active young men, age category: 18–25 years	↔ non-significant effect on basal BDNF content; ↑ significantly increase serum BDNF concentration immediately post-test	Blood parameter (BDNF)
**Sadowska-Krępa et al. [43]**	Randomized, double-blind, placebo-controlled, parallel-groups study	Green tea extract (GTE)	One 250 mg GTE capsule contained 245 mg polyphenols, including 200 mg catechins	Two capsules once daily	Six weeks	N/A	16 healthy, physicallyactive young men, age category (18–25 years)	↔ non-significant effect on BDNF	Blood parameter (BDNF)

Abbreviations: Not applicable (N/A), epigallocatechin gallate (EGCG), cocoa flavanol (CF), placebo (PLA), functional magnetic resonance imaging (FMRI) based on blood oxygenation level-dependent (BOLD), stroke volume (SV), cerebral blood flow (CBF), oxygenated haemoglobin (oxy-HB), deoxygenated hemoglobin (deoxy-HB), continuous-wave near-IR spectroscopy (CW-NIRS), brain-derived neurotrophic factor (BDNF).

**Table 3:** Methodological quality of the studies with (poly)phenol-rich supplementation assessed with the PEDro scale.

	Items	File et al. [41]	Francis et al. [45]	Spaak et al. [52]	Kennedy et al. [46]	Wightman et al. [47]	Wightman et al. [48]	Massee et al. [49]	Wightman et al. [36]	Alharbi et al. [37]	Decroix et al. [50]	Lamport et al. [38]	Dietz et al. [39]	Haskell-Ramsay et al. [51]	Sadowska-Krepa et al. [42]	Karabay et al. [40]	Sadowska-Krepa et al. [43]
1	Eligibility criteria were specified	+	+	+	+	+	+	+	+	+	+	+	+	+	+	+	+
2	Subjects were randomly allocated to groups (in a crossover study, subjects were randomly allocated an order in which treatments were received)	+	+	+	+	+	+	+	+	+	+	+	+	+	+	+	+
3	Allocation was concealed	+	+	-	+	+	+	+	+	+	+	+	+	+	+	+	+
4	The groups were similar at baseline regarding the most important prognostic indicators	+	+	+	+	+	+	+	+	+	+	+	+	+	+	+	+
5	There was blinding of all subjects	+	+	+	+	+	+	+	+	+	+	+	+	+	+	+	+
6	There was blinding of all therapists who administered the therapy	+	+	-	+	+	+	+	+	+	+	+	+	+	+	+	+
7	There was blinding of all assessors who measured at least one key outcome	−	−	−	−	−	−	−	−	−	−	−	−	−	−	−	−
8	Measures of at least one key outcome were obtained from more than 85% of the subjects initially allocated to groups	+	+	+	+	+	+	+	+	+	+	+	+	+	+	+	+
9	All subjects for whom outcome measures were available received the treatment or control condition as allocated or, where this was not the case, data for at least one key outcome was analyzed by “intention to treat”	+	+	+	+	+	+	+	+	+	+	+	+	+	+	+	+
10	The results of between-group statistical comparisons are reported for at least one key outcome	+	+	+	+	+	+	+	+	+	+	+	+	+	+	+	+
11	The study provides both point measures and measures of variability for at least one key outcome	+	+	+	+	+	+	+	+	+	+	+	+	+	+	+	+
	Total score	9	9	7	9	9	9	9	9	9	9	9	9	9	9	9	9
